# NR5A2 promotes malignancy progression and mediates the effect of cisplatin in cutaneous squamous cell carcinoma

**DOI:** 10.1002/iid3.1172

**Published:** 2024-02-15

**Authors:** Wang Ye, Cao Ya‐xuan, Tang Shan‐shan, Long Qiu, Ma Ting, Chen Shao‐jie, Cao Yu

**Affiliations:** ^1^ School of Clinical Medicine Guizhou Medical University Guiyang China; ^2^ Department of Dermatology Affiliated Hospital of Guizhou Medical University Guiyang China; ^3^ Department of Hepatobiliary Surgery Affiliated Hospital of Guizhou Medical University Guiyang China

**Keywords:** cisplatin, cutaneous squamous cell carcinoma, metastasis, NR5A2, proliferation, Wnt/β‐catenin signaling pathway

## Abstract

**Introduction:**

Nuclear receptor subfamily five group A member two (NR5A2) plays a key role in the development of many tumor types, while it is uncertain in cutaneous squamous cell carcinoma (cSCC). The aim of this work was to determine the role of NR5A2 in cSCC proliferation, and to determine whether NR5A2 mediates the effect of cisplatin in cSCC.

**Methods:**

We performed a systematic study of existing data and conducted a preliminary bioinformatics analysis of NR5A2 expression in cSCC using bioinformatics databases. Immunohistochemical staining was performed on cSCC tissues of seven patients to study NR5A2 expression. NR5A2 expression was examined in human keratin‐forming cells (HaCaT) and human cSCC cells (A431, Colo‐16, SCL‐1, SCL‐2, and HSC‐5). Stable A431 and SCL‐2 cell lines consisting of sh‐RNA‐NR5A2 were constructed to detect changes in cell proliferation, cell cycle, apoptosis, and to determine the key proteins in the Wnt/β‐catenin pathway. We also investigated changes in the effects of cisplatin on cSCC cells by CCK‐8, clone formation assay, and Flow apoptosis assay after NR5A2 knockdown.

**Results:**

NR5A2 showed enhanced expression in cSCC tissues than in healthy tissues. Downregulation of NR5A2 in cSCC cells led to the formation of a less malignant phenotype. In contrast, the proliferative capacity of the cSCC cells was enhanced posttreatment with RJW100, an NR5A2 agonist. Additionally, NR5A2 knockdown led to a decrease in the expression level of the proteins in the Wnt/β‐catenin pathway, and this inhibition was reversed by LiCl and recombinant antibody, Wnt3a. Moreover, NR5A2 knockdown resulted in diminished proliferative capacity and increased apoptotic cells after the addition of cisplatin.

**Conclusion:**

NR5A2 plays a crucial role in the progression of cSCC, and the Wnt/β‐catenin signaling pathway may be involved in the regulation of NR5A2‐mediated cSCC. Knockdown of NR5A2 enhanced both the proliferation inhibiting and apoptosis promoting effects of cisplatin on cSCC.

## Graphical abstract

Nuclear receptor subfamily five group A member two (NR5A2) has a critical role in the occurrence and progression of cutaneous squamous cell carcinoma (cSCC), and it may be played via the Wnt/β‐catenin, at the same time, knockdown of NR5A2 expression mediates the effect of cisplatin in cSCC.

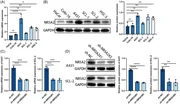

## 1 INTRODUCTION

Cutaneous squamous cell carcinoma (cSCC) features high aggressiveness and a high recurrence rate.^1^ The prevalence of cSCC has grown in recent years because of the increasing age and high level of exposure to ultraviolet radiation.^2^ cSCC often occurs in the cutaneous stratum corneum of the exposed areas such as the head, face, and neck, which is an important part of the head and neck squamous cell carcinoma (HNSCC). The early clinical signs of which are not evident, therefore, some patients already show signs of distant metastases at the time of diagnosis, and the prognosis is often poor in these patients.^3^ The pathogenesis of cSCC is not completely understood, and thus, no targeted drugs are currently available. Therefore, it is crucial to conduct research into the biological processes underlying cSCC development to identify treatment targets and prognostic indicators.

NR5A2 is a key orphan receptor belonging to the NR5A2 subfamily. NR5A2 can maintain the pluripotency of stem cells during embryonic development, is an important regulator of embryonic development, and can regulate the function of metabolism‐related genes.^4^ Recent research findings have confirmed the involvement of NR5A2 in the genesis and development of multiple tumor types, including esophageal and lung squamous tumors; breast, pancreatic, and gastric cancers; and hepatocellular carcinomas.^5^, ^6^ In one study of lung adenocarcinomas, elevated expression levels of NR5A2 negatively correlated with overall survival,^7^ while in another study of non‐small cell lung cancer, elevated expression levels of NR5A2 positively correlated with distant metastatic events.^8^ A study on hepatocellular carcinoma showed that the downregulation of NR5A2 expression using gene silencing technology inhibits the process of cell proliferation.^9^ Additionally, NR5A2 is also highly expressed in adriamycin‐resistant breast cancer cells, and the silencing of NR5A2 expression leads to the reversal of cellular resistance.^10^, ^11^ Moreover, NR5A2 has also been shown to be crucial for cell invasion and migration. For instance, NR5A2 has been found to contribute to the malignancy of pancreatic cancer through an increase in the translational activity of β‐catenin and expression of its target genes.^12^ Reorganization of the actin cytoskeleton and cleavage of E‐cadherin also enhance the malignant behavior of NR5A2‐overexpressing breast cancer cells.^13^ In summary, these findings suggest that the inhibition of NR5A2 may be an effective strategy for slowing down the development as well as progression of many tumor types.

Numerous studies have shown that NR5A2 may play a role in the development of resistance to anticancer drugs. In a study by Yang et al.,^14^ NR5A2 was expressed at high levels in glioma tissues, and the overexpression of NR5A2 in temozolomide (TMZ)‐resistant glioma cells increased their resistance to the drug while the knockdown of NR5A2 reduced their resistance to the drug. Similarly, in a study by Qiao et al.^15^ on breast cancer, NR5A2 acted synergistically with NCOA3 and attenuated the process of mastocytosis via the upregulation of NRF2, which in turn induced resistance to BET inhibitors (BETi); however, the use of a small‐molecule inhibitor of NR5A2 enhanced the activity of BETi both in vivo and in vitro, thereby enhancing its anticancer effects in breast cancer cells. Based on the findings of these studies, it is also interesting to study the effect of NR5A2 on the action of chemotherapeutic drugs.

Although previous studies have demonstrated that NR5A2 possesses an oncogenic role, some researchers have suggested the opposite. Gkikas et al.^16^ found that a high expression of NR5A2 often predicts a favorable prognosis for patients with glioblastoma and neuroblastoma tumors. Their study also revealed a role for NR5A2 in nerve‐related malignant tumors, and that the growth of this type of tumor was significantly inhibited by the use of an agonist for NR5A2. Although the overexpression of NR5A2 accelerates malignant processes such as proliferation and invasion in pancreatic cancer, the loss of NR5A2 expression limits recovery from injury in pancreatitis, which in turn increases the risk of pancreatic ductal adenocarcinoma.^17^

The potential function of NR5A2 in cSCC is uncertain. Therefore, in this study, we determined the role of NR5A2 in the proliferation, cell cycle progression, cell migration, and invasion of cSCC cells. Additionally, we also determined the role of NR5A2 in regulating the effect of cSCC cells to chemotherapeutic drugs.

## 2 MATERIALS AND METHODS

### 2.1 Ethical approval

The study was conducted in accordance with the Declaration of Helsinki, and the study protocol was approved by the Ethics Committee of the Affiliated Hospital of Guizhou Medical University (approval number: 2023650).

### 2.2 Assessment of NR5A2 expression in cSCC tissues

The expression of RNA datasets for HNSCC and matched healthy tissues were obtained from The Cancer Genome Atlas (TCGA; https://portal.gdc.com) and Genotype‐Tissue Expression (GTEx; https://www.gtexportal.org/home/) were preprocessed using the “limma” package in R STUDIO. HNSCC was divided into two groups based on the differences in the mRNA levels of NR5A2 and visualized using box plots obtained with the “boxplot” package. The relationship between NR5A2 expression level and HNSCC grade was determined using the cancer data analysis portal of the University of Alabama at Birmingham (UALCAN; https://ualcan.path.uab.edu/index.html). The differences in the protein levels of NR5A2 between the tumor and healthy tissues were also analyzed using the Human Protein Atlas (HPA; https://www.proteinatlas.org/).

### 2.3 Immunohistochemical (IHC) staining of samples from patients with cSCC

The tissue removed during surgery was collected from seven patients with cSCC while ensuring that the patients faced no additional harm. Seven sets of matched cancer and para‐cancerous tissues were double‐checked by two pathologists using hematoxylin and eosin staining. Paraffin‐embedded tissues were sectioned and subjected to antigen repair, followed by endogenous enzyme elimination, incubation with NR5A2 antibody for 12 h at 4°C (Abclonal Technology; # A16584, 1:100), observation for color change in the tissues, and photography of the tissues.

### 2.4 Cell culture

Cell lines of human keratin‐forming cells (HaCaT) and cSCC cells (A431, Colo‐16, SCL‐1, SCL‐2, and HSC‐5) were purchased from Procell Life Science & Technology Co. Ltd. The cells were cultured in a medium comprising a mixture of DMEM and FBS at a ratio of 9:1, and placed in an incubator set at 37°C and containing 5% carbon dioxide and 95% air.

### 2.5 Stable sh‐NC and sh‐NR5A2 cell line construction

A lentiviral vector (HanBio Biotechnology) was used to construct stable cell lines of A431 and SCL‐2 cells consisting of short hairpin RNA‐NR5A2 (sh‐NR5A2), and negative control (sh‐NC). The oligonucleotide coding sequences for sh‐NR5A2 (sh‐1, sh‐2) were 5′‐GCAGCAGACAGAGAAATTT‐3′ and 5′‐AAACACAGAAGTCGCATTCAA‐3′, respectively, and for sh‐NC it was 5′‐TTCTCCCCGAACGTGTCACGT‐3′. The cells were digested with trypsin (Invitrogen Gibco). The next day, the cells were inoculated into six‐well plates at a density of 2.5 × 10^6^ cells/well and transfected with the lentivirus. Two days later, the cells were screened by treatment with puromycin at a concentration of 1.5 µg/mL.

### 2.6 Pharmacological treatments

In studies on the role of NR5A2 agonizts, the cells were treated with RJW100 (25 µM, MedChemExpress; HY‐131445) and incubated for 24 h in the incubator, then followed by other experiments. In the studies of recombinant human Wnt3a protein (Proteintech Group; HZ‐1296), it was added at a concentration of 100 ng/mL, and the cells were incubated for 6 h in the incubator, then followed by other experiments.

### 2.7 Real‐time quantitative PCR

Trizol (Solarbio Life Science) was added to the cells to extract the total RNA, which was reverse‐transcribed using the PrimeScript RT Master Mix Kit (Takara). cDNA amplification was performed using SYBR Premix Ex Taq (Takara). The housekeeping gene GAPDH was used as the control, and gene expression was calculated relatively using the formula: 2^−^^ΔΔCt^. The primer sequences used in the PCR assay were as follows: NR5A2 (5′ to 3′, forward: CTTTGTCCCGTGTGTGGAGAT; reverse: GTCGGCCCTTACAGCTTCTA); GAPDH (5′ to 3′, forward: GGAGCGAGATCCCTCCAAAAT; reverse: GGCTGTTGTCATACTTCTCATGG).

### 2.8 Western blot analysis

Cellular proteins were collected using RIPA lysate (Solarbio Life Science) containing 1% protease inhibitor (Solarbio Life Science). The proteins were electrophoresed on 10% SDS‐PAGE (Epizyme) and transferred to a PVDF membrane. To prevent nonspecific binding, the PVDF membrane was incubated in 5% skimmed milk for 1.5 h at 20°C. Subsequently, the primary antibodies were added at a moderate dilution and the PVDF membrane was incubated overnight, with shaking, in a refrigerator at 4°C. The corresponding secondary antibodies were then added and the membrane was incubated for 1 h at 20°C, treated with ECL solution (Meilunbio) for 1 min, and detected on the Tanon‐5200 automated chemiluminescence system (Gene Company Limited). The grayscale values were then counted by ImageJ software (v. 2.1.0) (Bethesda Softworks). The antibodies used in the assay were as follows: NR5A2 (#22460‐1‐AP, 1:1000), β‐catenin (#51067‐2‐AP, 1:5000), cyclin D1 (#26939‐1‐AP, 1:5000), c‐MYC (#10828‐1‐AP, 1:3000), E‐cadherin (#20874‐1‐AP, 1:1000), Vimentin (#10366‐1‐AP, 1:3000), N‐cadherin (#22018‐1‐AP, 1:5000), Slug (#12129‐1‐AP, 1:2000), Snail (#13099‐1‐AP, 1:7000), GAPDH (#10494‐1‐AP, 1:5000), Bax (#60267‐1‐Ig，1:5000), Bcl‐2 (#60178‐1‐Ig, 1:10,000), and HRP‐conjugated AffiniPure Rabbit Anti‐Goat IgG (#SA00001‐2, 1:6000). All antibodies were purchased from Proteintech Group.

### 2.9 Cell proliferation assay

The CCK‐8 assay kit (Meilunbio) was used to detect cell proliferation. sh‐NC and sh‐NR5A2 cells were inoculated at a density of 4000 cells/100 μL/well in a 96‐well plate, and incubated at 37°C for 1–5 days. Following incubation, CCK‐8 reagent was added at a concentration of 10 μL/well, as per the manufacturer's instructions. The cells were incubated at 37°C for 1.5 h, and the absorbance of the solution was measured at 450 nm using an Absorbance Microplate Reader (Molecular Devices). The experiment was repeated three times, independently.

### 2.10 Clone formation experiments

sh‐NC and sh‐NR5A2 cells in the log phase of growth were digested into single‐cell suspensions and inoculated in 6‐well plates at a density of 1000 cells/well. Every 2 days, the medium was renewed and the cells were cultured for 12 days. Cells were then fixed and stained, and the clone count was obtained.

### 2.11 Cell cycle analysis

Cell cycle analysis was performed using the cell cycle kit (meilunbio). After transfection with sh‐nr5a2 and sh‐nc, cells were fixed by a solution consisting of 70% anhydrous ethanol and 30% pbs for 7 h. The cells were then mixed with a solution of RNase a/propidium iodide (10:1), gently resuspended, and incubated at 37°C for 30 min. Subsequently, the cells were subjected to flow cytometry (BD Biosciences) analysis.

### 2.12 Wound healing assay

Two straight lines were drawn at the back of the culture plate using a marker. The transfected cells were cultured into the plate individually and incubated until the cell growth density reached 90% confluence, and then treated with mitomycin C (1 µg/mL, MedChemExpress, HY‐13316) for 1 h to inhibit the proliferative effect of cells. Another set of lines was drawn on the cells, perpendicular to those at the back of the plate, using 200‐µL tips. The unbound cells were washed off, and the cells that remained on the plate were transferred to a DMEM medium containing 2% FBS. Subsequently, the regions where the lines intersected were photographed under a microscope. After 48 h, these regions were again observed under a microscope and photographed.

### 2.13 Cell invasion assay

The Matrigel (BD Biosciences) was diluted in DMEM at a ratio of 1:5, and 40 µL of the Matrigel was added to the upper chamber of the transwell and allowed to solidify to form a thin layer of gel. Once the Matrigel solidified, the cells were prepared to a single‐cell suspension and centrifuged. The cells were then re‐suspended and the concentration of the suspension was adjusted to 3 × 10^5^ cells/mL using a culture medium free of FBS. About 200 μL of the cell suspension was added in the upper chamber, and a culture medium with 15% FBS was added in the lower transwell. The cells were incubated at 37°C for 48 h, transwell chambers were fixed using 4% paraformaldehyde solution and then stained using 0.1% crystal violet solution. The cells and the Matrigel in the upper chamber were then removed. The cells in five randomly selected microscopic fields were counted, and the mean cell count was calculated.

### 2.14 Cell apoptosis assay

The cells were resuspended according to the manufacturer's instructions (Multisciences), and 5 μL of annexin V‐APC staining solution was added, followed by 10 μL of 7‐ADD staining solution. The cell suspension was incubated in the dark for 30 min at 37°C, and subsequently subjected to flow cytometry analysis.

### 2.15 Cisplatin treatment assay

Cisplatin (Solarbio Life Science) was dissolved in 1X PBS to obtain a concentration of 5 µM and stored at −20°C. The cells were treated with a cisplatin concentration gradient, incubated for 48 h, and subjected to CCK‐8 assay. The absorbance of the cell suspension was measured and the survival rate was calculated. For clone formation experiments in cells treated with drugs, the cells were treated with a 2 µM concentration of cisplatin and incubated for 48 h, followed by changing the conventional medium to continue the culture. For the apoptosis assay, posttreatment with 2 µM of cisplatin and incubation for 48 h, the cells were subjected to flow cytometry analysis to detect the rate of apoptosis.

### 2.16 Statistical analysis

All experiments were repeated at least three times, and the data are reported as mean ± SD. The data were subjected to a normal distribution test by Shapiro–Wilk. The significance analyzes were performed by one‐way analysis of variance (ANOVA), Unpaired *t*‐test, and two‐way ANOVA using GraphPad Prism 9.0 software (GraphPad Software). In all figures, the *p*‐values are denoted as ****p < .0001, ***p < .001, **p < .01, *p < .05, and ^ns^ *p* ≥ .05.

## 3 RESULTS

### 3.1 NR5A2 expression was significantly higher in cSCC tissues than in healthy noncancerous tissues

**Figure 1:**
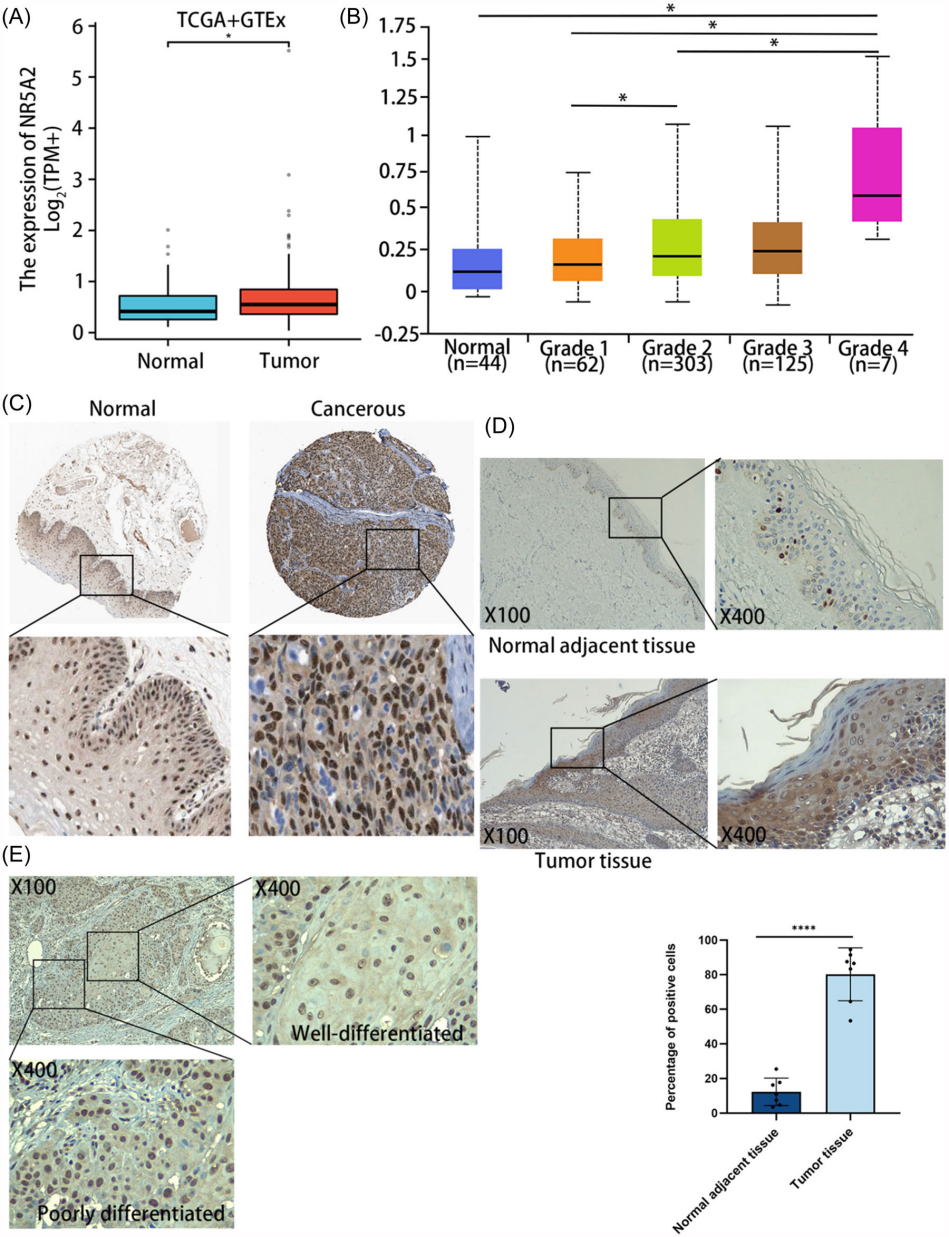
Nuclear receptor subfamily five group A member two (NR5A2) expression is higher in cutaneous squamous cell carcinoma (cSCC) tissues compared to the noncancerous tissues. (A) NR5A2 gene expression in healthy and head and neck squamous cell carcinoma (HNSCC) tissues. (B) NR5A2 gene expression in HNSCC based on tumor grade. (C) NR5A2 gene expression in healthy and HNSCC tissues. (D) NR5A2 expression in clinical samples of healthy and cSCC tissues. (E) NR5A2 expression in clinical samples of different differentiated tissues of cSCC. (*p < .05; ****p < .00001).

Bioinformatics analysis of NR5A2 mRNA expression across the TCGA and GTEx databases revealed that the expression level of NR5A2 was higher in HNSCC tissues than in healthy tissues (Figure 1A). Similar results were obtained in the NR5A2 protein expression analysis in the HPA database (Figure 1C). In HNSCC tissues, the expression level of NR5A2 was closely associated with the tumor grade; the higher the tumor grade, the higher the expression level of NR5A2 (Figure 1B). IHC analysis revealed that cSCC tissues stained significantly darker compared to healthy tissues (Figure 1D). And in cSCC, NR5A2 expression was mainly localized in the nucleus. Compared to well‐differentiated keratinized strains, the expression of NR5A2 was higher in poorly differentiated tissues (Figure 1E).

### 3.2 NR5A2 knockdown reduced the rate of proliferation in A431 and SCL‐2 cells

**Figure 2:**
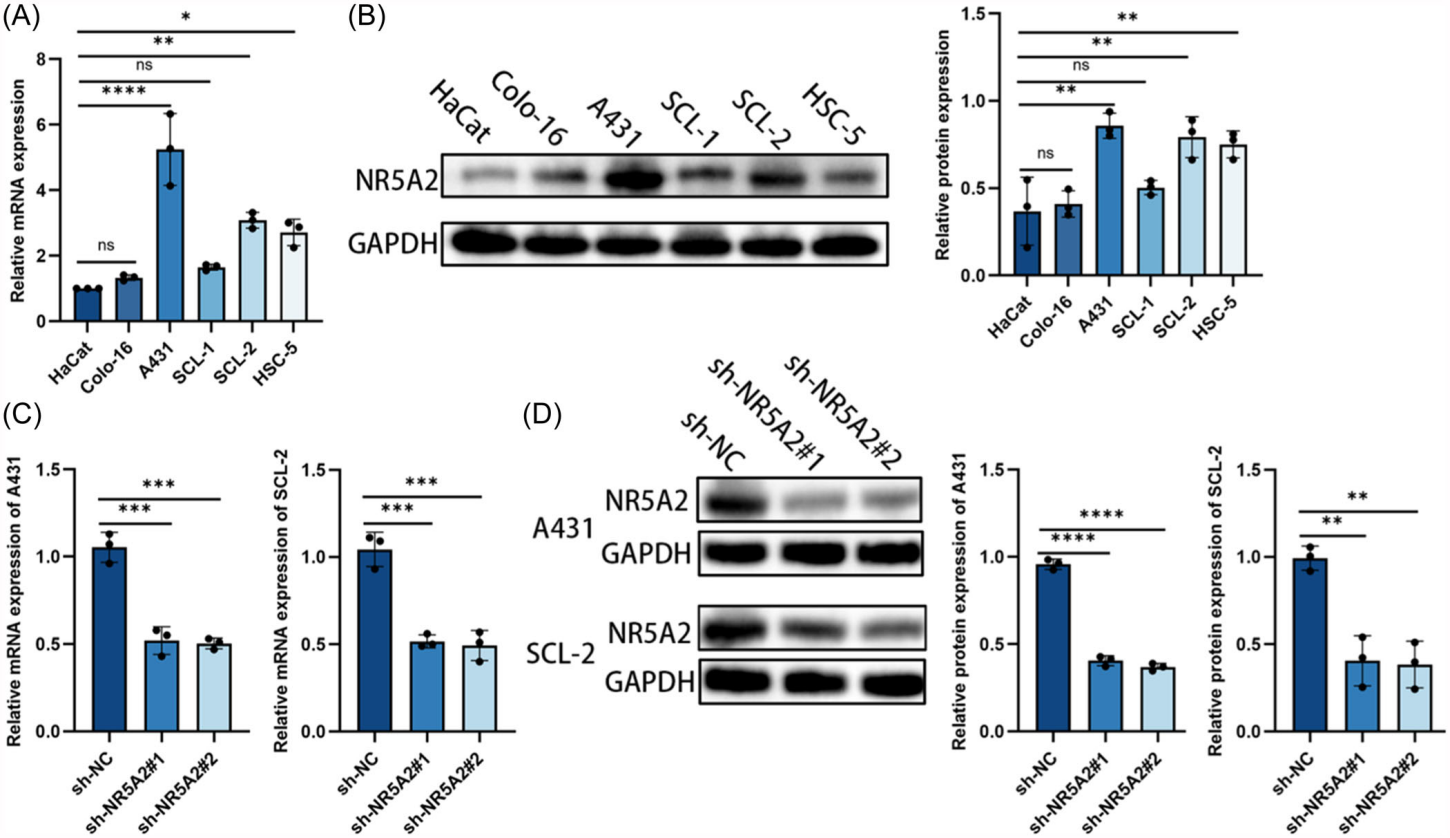
Expression of nuclear receptor subfamily five group A member two (NR5A2) in HaCat and cutaneous squamous cell carcinoma (cSCC) cells. (A, B) mRNA and protein expression of NR5A2 in normal keratin‐forming cells (HaCaT) and cSCC cells (Colo‐16, A431, SCL‐1, SCL‐2, and HSC‐5). (C, D) Changes in NR5A2 mRNA and protein expression in A431 and SCL‐2 cells after transfection with sh‐NR5A2. (One‐way analysis of variance [ANOVA] with Tukey's multiple comparisons test was performed for A–C. Two‐way ANOVA with Tukey's multiple comparisons test was performed for D.*p < .05; **p < .01; ***p < .001; ****p < .00001, ^ns^ *p* ≥ .05).

**Figure 3:**
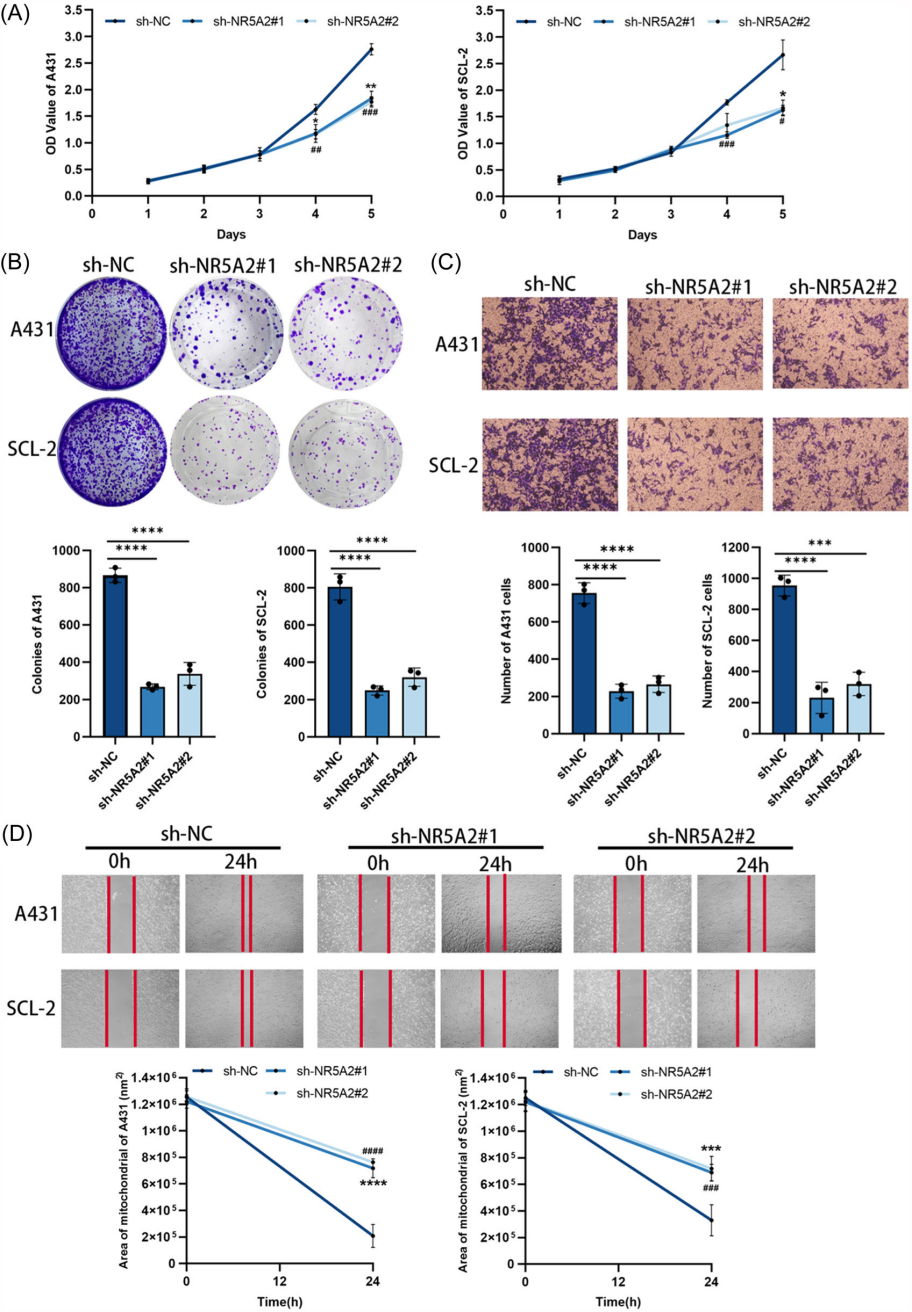
Knockdown nuclear receptor subfamily five group A member two (NR5A2) inhibits the proliferation, clone formation, migration, and invasion capacities of cutaneous squamous cell carcinoma (cSCC) cells. (A) The absorbance of A431 and SCL‐2 cells with downregulated NR5A2 at 450 nm. (B) Effect of NR5A2 downregulation on the clone formation ability of A431 and SCL‐2 cells. (C) Effect of NR5A2 downregulation on the migration capacity of A431 and SCL‐2 cells. (D) Effect of NR5A2 downregulation on the invasiveness of A431 and SCL‐2 cells. (Two‐way analysis of variance [ANOVA] with Tukey's multiple comparisons test was performed for A and D. One‐way ANOVA with Tukey's multiple comparisons test was performed for B and C. *p < .05; **p < .01; ***p < .001; ****p < .00001; ^####^ *p* < .00001 vs. sh‐NC group).

Both qRT‐PCR and Western blot experiments revealed that the gene as well as protein levels of NR5A2 were higher in A431, SCL‐2, and HSC‐5 cells compared to HaCat (Figure 2A,B). Subsequent experiments demonstrated that NR5A2 expression was downregulated in A431 and SCL‐2 cells posttransfection with two kinds of sh‐NR5A2 (Figure 2C,D). A significant decrease in cell proliferation was noted in A431 and SCL‐2 cells transfected with sh‐NR5A2 on Day 4 posttransfection (Figure 3A); these cells also showed a reduction in the number of clones (Figure 3B). In the transwell assays, cells with NR5A2 knockdown demonstrated reduced invasive capacity compared to sh‐NC (Figure 3C). Wound healing assays showed that sh‐NR5A2 had a lower migratory capacity compared to sh‐NC (Figure 3D).

### 3.3 Increased expression level of NR5A2 promoted cSCC cell proliferation

**Figure 4:**
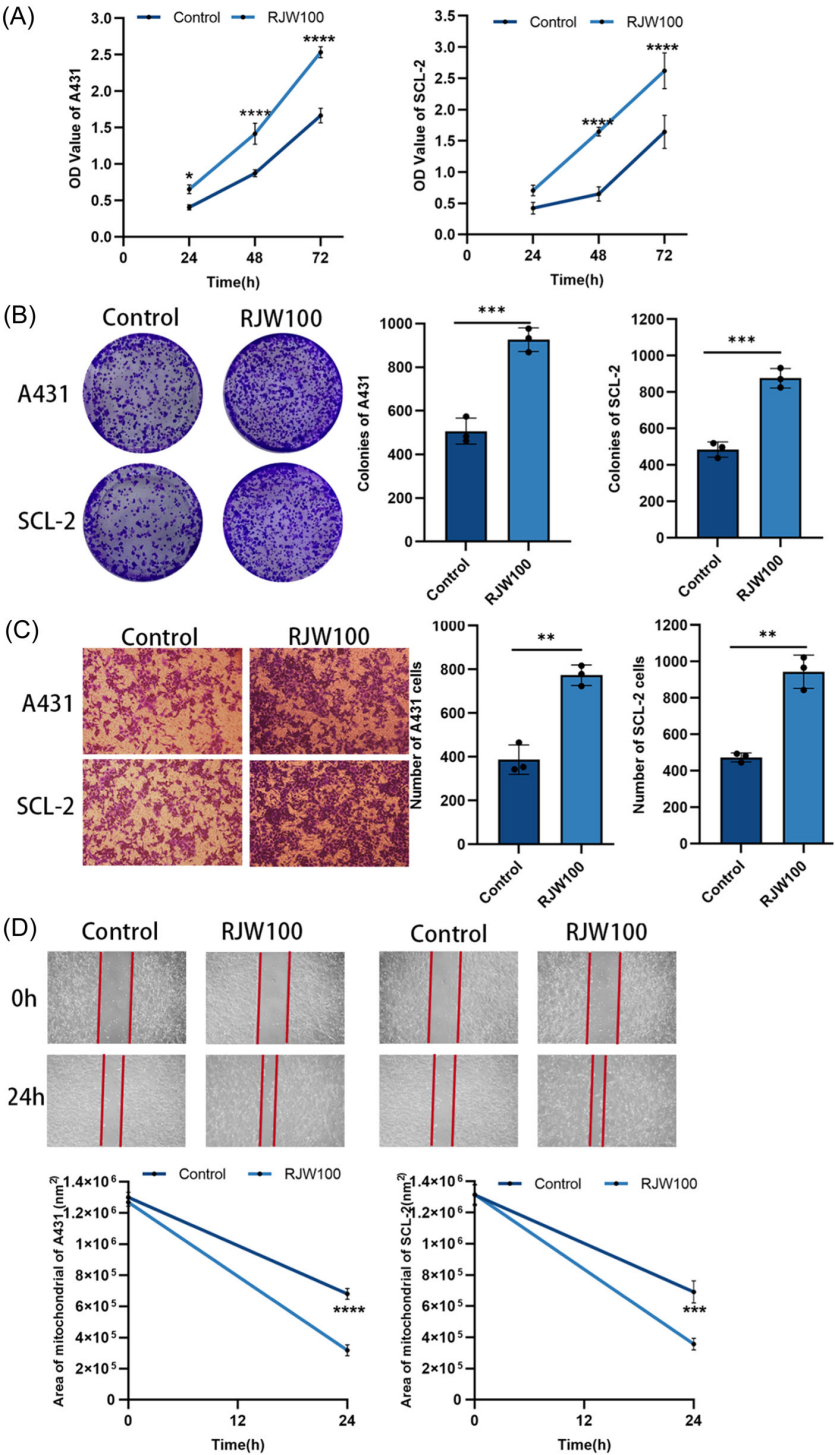
Increased expression of nuclear receptor subfamily five group A member two (NR5A2) promotes cutaneous squamous cell carcinoma (cSCC) cell proliferation. (A) Absorbance of A431 and SCL‐2 cells treated with the NR5A2 agonist, RJW100 (at 100 ng/mL, for 24 h), at 450 nm. (B) NR5A2 agonist RJW100 affects the clone formation ability of A431 and SCL‐2 cells. (C) Effect of NR5A2 agonist RJW100 on the migration capacity of A431 and SCL‐2 cells. (D) Effect of NR5A2 agonist RJW100 on the invasive ability of A431 and SCL‐2 cells. (Two‐way analysis of variance with Šidák's multiple comparisons test was performed for A and D. Unpaired *t*‐test was performed for B and C. *p < .05; **p < .01; ***p < .001; ****p < .00001).

The proliferative capacity of A431 and SCL‐2 cells was significantly enhanced after treatment with the NR5A2 agonist, RJW100 (Figure 4A). The number of clones was also higher among the A431 and SCL‐2 groups compared to that of the control group (Figure 4B). Additionally, transwell and wound‐healing assays also demonstrated that A431 and SCL‐2 cells had stronger migratory and invasive capacities compared to that of the control group (Figure 4C,D).

### 3.4 NR5A2 knockdown led to apoptosis and G0/G1 phase cell cycle block in cSCC cells and affected the expression of epithelial‐mesenchymal transition‐related proteins

**Figure 5:**
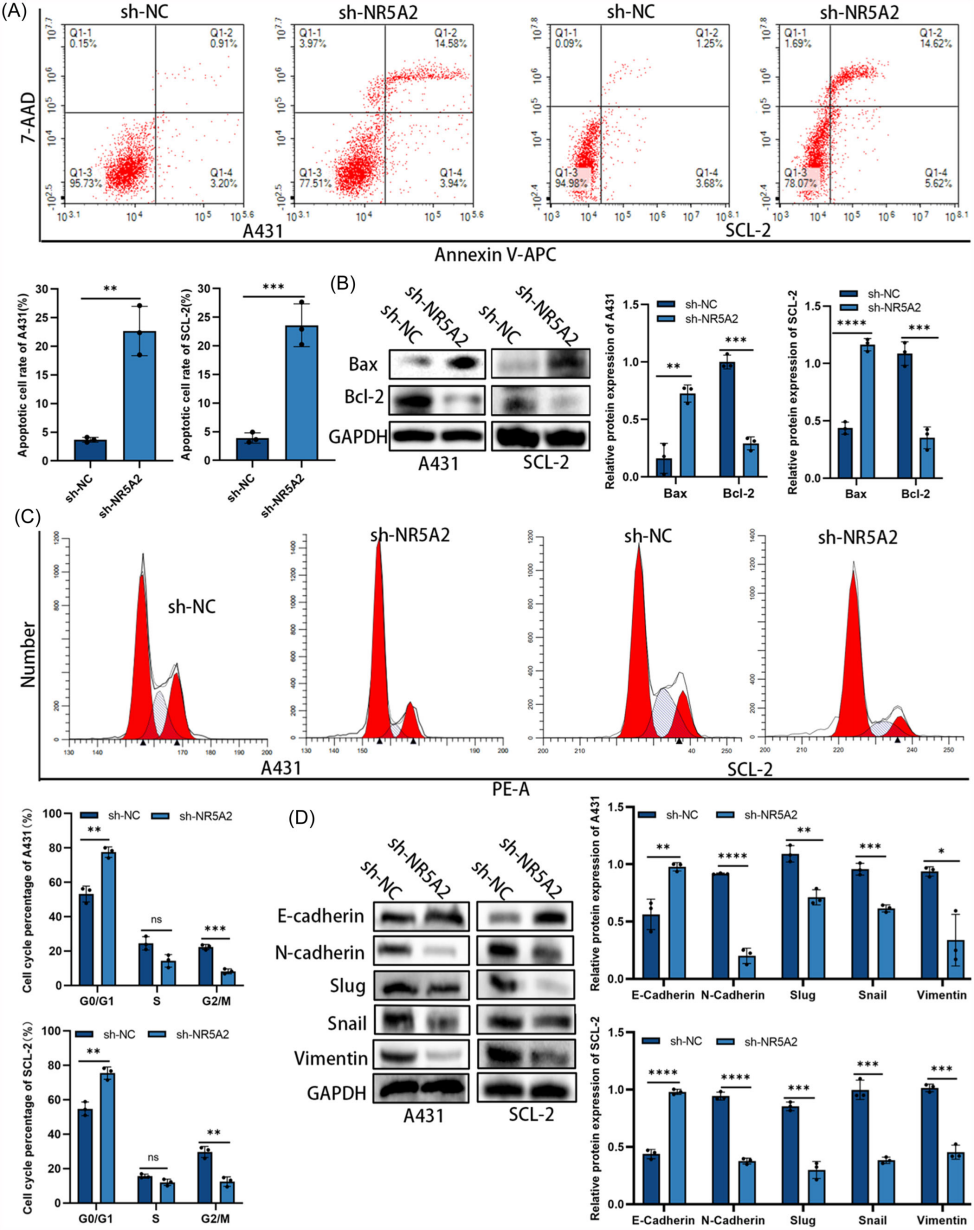
Knockdown of nuclear receptor subfamily five group A member two (NR5A2) leads to apoptosis and G0/G1 phase cell cycle block in cutaneous squamous cell carcinoma (cSCC) cells and affects the expression of epithelial‐mesenchymal transition‐related proteins. (A) Effect of NR5A2 downregulation on the rate of apoptosis of A431 and SCL‐2 cells. (B) Effect of NR5A2 downregulation on the protein expression levels of Bax and Bcl‐2. (C) Effect of NR5A2 downregulation on A431 and SCL‐2 cell cycle progression. (D) Effect of NR5A2 downregulation on the expression levels of key epithelial‐mesenchymal transition (EMT)‐related proteins. (Unpaired *t*‐test was performed for A, C, and D. Two‐way analysis of variance with Šidák's multiple comparisons test was performed for B. *p < .05; **p < .01; ***p < .001; ****p < .00001).

NR5A2 knockdown led to a ~4X increase in the rate of apoptosis in A431 and SCL‐2 cells compared with that in the sh‐NC group (Figure 5A). Examination of the apoptosis‐related proteins Bax and Bcl‐2 revealed that the expression of Bax was upregulated after NR5A2 knockdown, while that of Bcl‐2 decreased (Figure 5B). Cell cycle analysis also revealed that a greater proportion of cells were found in the G0/G1 phase after the downregulation of NR5A2 (Figure 5C).

Since NR5A2 possesses the ability to inhibit cell migration, we investigated whether NR5A2 affects epithelial‐mesenchymal transition (EMT) in A431 and SCL‐2 cells. We found that the expression levels of the key proteins associated with EMT changed after NR5A2 knockdown. We found that the expression level of E‐cadherin increased while that of N‐cadherin, vimentin, snail, and slug decreased after NR5A2 downregulation (Figure 5D).

### 3.5 NR5A2 knockdown inhibited cell proliferation and migration by inhibiting the Wnt/β‐catenin signaling pathway

**Figure 6:**
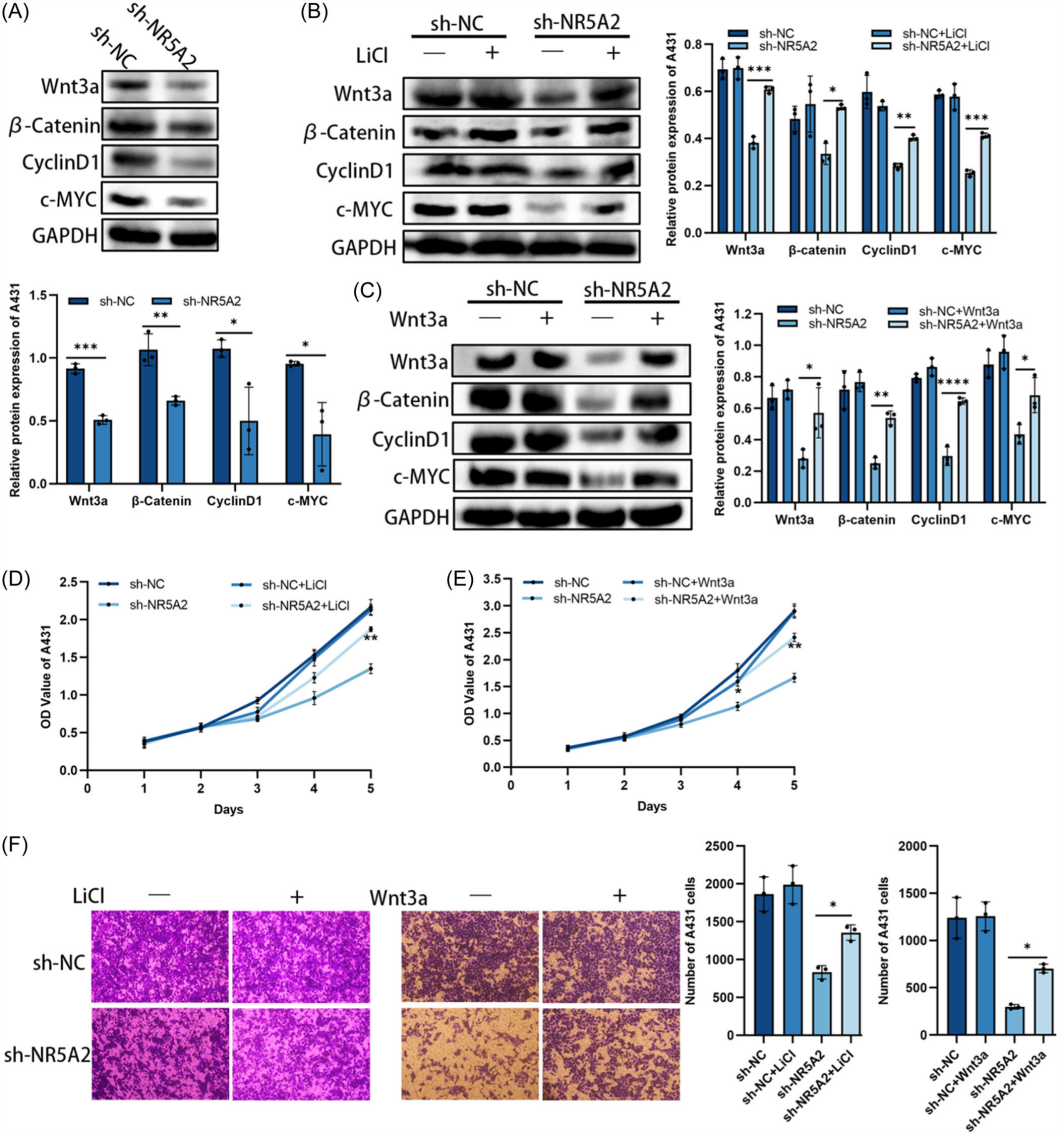
Nuclear receptor subfamily five group A member two (NR5A2) affects the proliferation and invasion of A431 cells via the Wnt/β‐catenin signaling pathway. (A) NR5A2 downregulation affects the protein levels of Wnt3a, β‐catenin, cyclin D1, and c‐MYC. (B) Effect of downregulation of NR5A2 on the protein levels of Wnt3a, β‐catenin, cyclin D1, and c‐MYC, in the presence or absence of LiCl. (C) Effect of downregulation of NR5A2 on the protein levels of Wnt3a, β‐catenin, cyclin D1, and c‐MYC, in the presence or absence of the recombinant protein of Wnt3a. (D, E) Effect of downregulation of NR5A2 on A431 cell proliferation, in the presence or absence of LiCl or the recombinant protein of Wnt3a. (F) Effect of downregulation of NR5A2 on A431 cell invasion ability, in the presence or absence of LiCl or the recombinant protein of Wnt3a. (Unpaired *t*‐test was performed for A. One‐way analysis of variance [ANOVA] with Tukey's multiple comparisons test was performed for B, C, and F. Two‐way ANOVA with Tukey's multiple comparisons test was performed for D and E. *p < .05; **p < .01; ***p < .001; ****p < .00001).

NR5A2 knockdown led to a decrease in the expression levels of β‐catenin, Wnt3a, cyclin D1, and c‐MYC (Figure 6A). To specifically test for the changes in the Wnt/β‐catenin signaling pathway in A431 cells transfected with sh‐NC and sh‐NR5A2, the A431 cells were treated with 20 mM of LiCl (an activator of the Wnt/β‐catenin pathway) and a recombinant protein of Wnt3a. Subsequently, the cells were assessed for changes in signaling, cellular proliferation, and cellular mobility. The results depicted in Figure 6B,C reveal that the Wnt/β‐catenin pathway, which was inhibited by NR5A2 knockdown, could be reversed by LiCl and the recombinant protein of Wnt3a. The CCK‐8 assay also demonstrated that the inhibition of cellular proliferation by NR5A2 could be restored by LiCl on Day 5 posttransfection (Figure 6D) while Wnt3a showed a similar effect on Day 4 (Figure 6E). In addition, the transwell assays demonstrated that LiCl and Wnt3a enhanced the invasiveness of A431 cells that originally showed reduced invasiveness owing to the effect of NR5A2 (Figure 6F).

### 3.6 NR5A2 knockdown affected the sensitivity of cSCC cells to cisplatin

**Figure 7:**
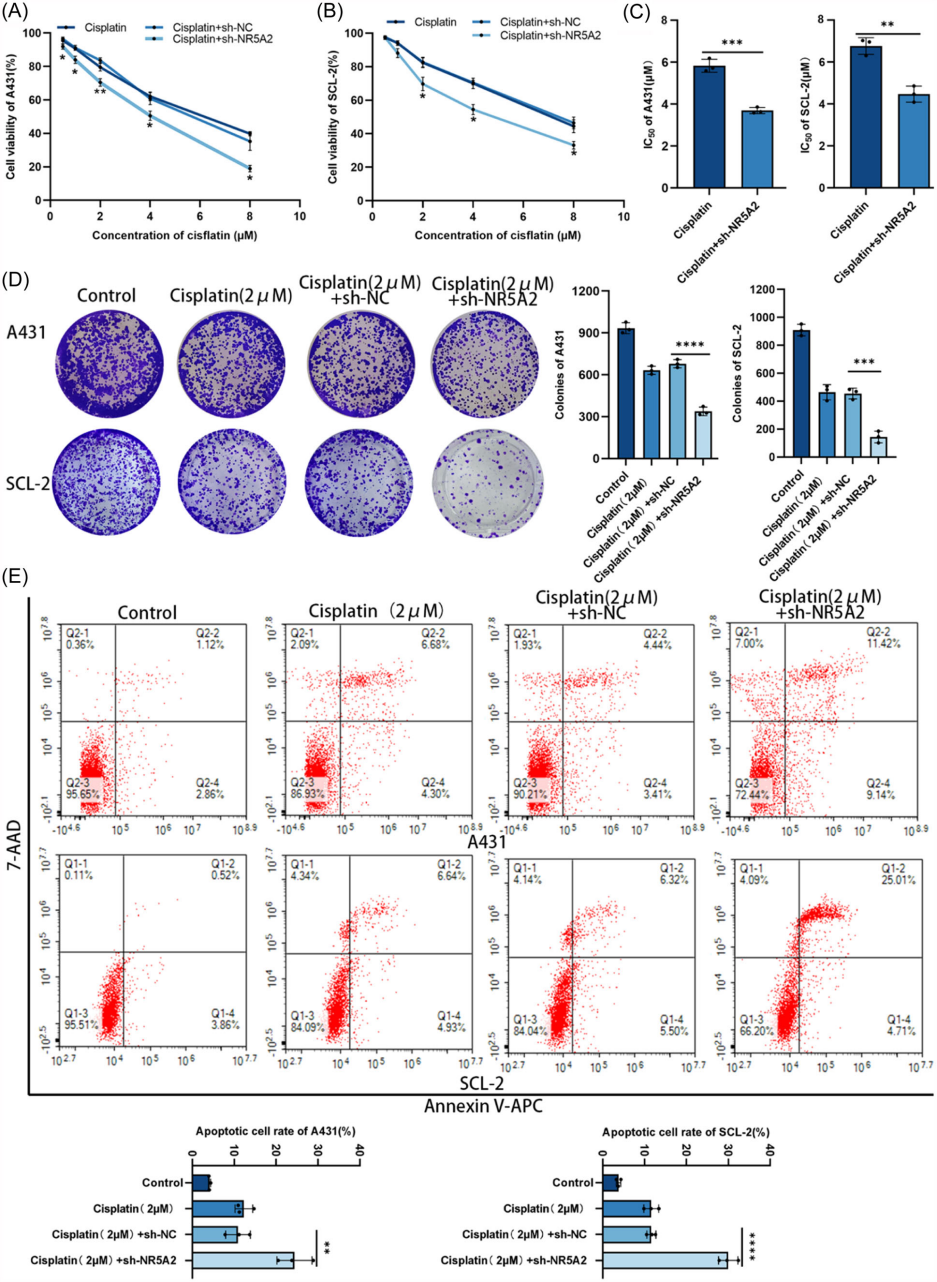
Downregulation of nuclear receptor subfamily five group A member two (NR5A2) affects the sensitivity of cutaneous squamous cell carcinoma (cSCC) cells to cisplatin. (A, B) Effect of NR5A2 downregulation on the viability of A431 and SCL‐2 cells treated with cisplatin. (C) Effect of NR5A2 downregulation on the half maximal inhibitory concentration (IC_50_) of cisplatin on the A431 and SCL‐2 cells. (D) Effect of NR5A2 downregulation on the formation of clones of A431 and SCL‐2 cells. (E) Effect of NR5A2 downregulation on the apoptosis rate of A431 and SCL‐2 cells. (Two‐way analysis of variance [ANOVA] with Tukey's multiple comparisons test was performed for A and B. Unpaired *t*‐test was performed for C. One‐way ANOVA with Tukey's multiple comparisons test was performed for D and E. **p < .01; ***p < .001; ****p < .00001).

To verify whether NR5A2 correlated with drug resistance to chemotherapeutic agents in cSCC, A431 and SCL‐2 cells transfected with sh‐NC and sh‐NR5A2 were treated with cisplatin. The results showed a significant reduction in cell viability in the presence of cisplatin after NR5A2 knockdown (Figure 7A,B). Additionally, the half‐maximal inhibitory concentration (IC_50_) of cisplatin was also lower after NR5A2 inhibition (Figure 7C).

Cloning experiments revealed that the group with suppressed NR5A2 expression had fewer clones compared to the cisplatin‐treated group (Figure 7D). Apoptosis analysis by flow cytometry analysis revealed that the rate of apoptosis was higher in the group with NR5A2 knockdown compared to the group treated with cisplatin alone (Figure 7E). These findings suggest that NR5A2 knockdown leads to an increase in the sensitivity of A431 and SCL‐2 cells to cisplatin.

## 4 DISCUSSION

Many studies have shown that NR5A2 plays a critical role in the progression of multiple tumor types. In this study, we have demonstrated that NR5A2 plays a significant role in the progression of cSCC. We found that NR5A2 is expressed at higher levels in poorly differentiated cSCC tissues than in well‐differentiated, contributing to increased cell proliferation and migration in cSCC. In addition, NR5A2 knockdown was found to downregulate the expression of the key proteins in the Wnt/β‐catenin pathway, suggesting that NR5A2 may promote cSCC progression via the Wnt/β‐catenin pathway.

Cobo et al.^16^ have shown that high expression of NR5A2 sensitizes pancreatic cells to inflammation, which in turn leads to differentiation of inflammatory cells towards tumor cells. However, at the same time, in mice, Nr5a2 deficiency leads to an unstable differentiation state of the mature pancreas, pancreatic migration to the ducts, and loss of regenerative capacity after acute pancreatitis.^17^ Thus, NR5A2 may play a regulatory role in cell differentiation that may be controversial. In our study, NR5A2 was highly expressed in poorly differentiated tumor tissues than in the well‐differentiated.

Previous research has shown that the Wnt/β‐catenin pathway acts as role in tumor development and progression.^18^ When the Wnt/β‐catenin pathway is activated in tumor cells, the transcription of its target genes is enhanced by the direct or indirect binding of β‐catenin to their promoters, leading to cell proliferation and migration.^19^ A study has shown that NR5A2 is a transcriptional co‐activator of β‐catenin, binding to β‐catenin through its C‐terminal ligand‐binding domain and maintaining the stability of the bond between β‐catenin and the target gene promoter, thereby promoting the transcription of the target gene.^20^ Several studies have shown that interference with the NR5A2‐mediated Wnt/β‐catenin pathway using pharmacological agents or via gene regulation can significantly inhibit tumor proliferation, migration, and invasion.^21^ In a study of gastric cancer, cancer cell proliferation was inhibited after NR5A2 silencing.^22^ In another study, the exogenous overexpression of microRNA‐381 for the obstruction of the function of the NR5A2‐mediated Wnt/β‐catenin signaling pathway led to an increase in the rate of apoptosis in colorectal cancer cells.^23^ Cpd3 and Cpd3d2, inhibitors of NR5A2, have also been proven to inhibit the malignant behavior of cancer cells by interfering with β‐catenin.^24^ In this study, we found that NR5A2 knockdown led to a decrease in the protein levels of β‐catenin, Wnt3a, cyclin D1, and c‐MYC in A431 cells, resulting in reduced cell proliferation, migration, and invasion. We also demonstrated that this reduction in cell proliferation, migration, and invasion could be reversed by LiCl, an activator of the Wnt/β‐catenin signaling pathway. Similarly, addition of Wnt3a recombinant protein to cells reversed the inhibitory effect of NR5A2 on cSCC cells. These findings suggest that NR5A2 promotes cSCC progression by mediating the Wnt/β‐catenin signaling pathway.

Several studies have focused on the function of NR5A2 in cell cycle progression. For instance, in a study of pancreatic cancer, activation of the BRD4 gene in cancer cells causes NR5A2 to be transcribed and upregulated, thus promoting pancreatic cancer progression.^25^ Other studies have shown that the silencing of NR5A2 expression blocks cells in the G0/G1 phase in colorectal cancer^23^ and glioma.^14^ Similar results were also obtained in our study in cSCC cells.

The role of NR5A2 in EMT has been evaluated in previous research. In EMT, epithelial cells acquire mesenchymal cell characteristics, giving the cells greater migratory and invasive properties, thus allowing the cancer cells to metastasize.^26^, ^27^ A previous study reported that NR5A2 knockdown in pancreatic cancer led to an increase in the expression level of E‐cadherin and a decrease in the expression levels of snail and vimentin, suggesting that NR5A2 is involved in the EMT process.^28^ Similar findings were also observed in our study. These findings confirm that NR5A2 plays an integral role in the EMT process in cSCC.

A previous study on breast cancer reported that NR5A2 expression was higher in the cancer tissues compared to healthy tissues. Additionally, higher levels of NR5A2 were also detected in adriamycin‐resistant breast cancer cells, and the overexpression of NR5A2 in breast cancer cells and transplanted tumors in nude mice led to an attenuation in the effects of adriamycin and cisplatin on the cancer cells.^29^ NR5A2 also promotes cell proliferation in hepatoblastoma, and the toxic effects of adriamycin on hepatoblastoma can be potentiated by the use of NR5A2 antagonists.^30^ Therefore, NR5A2 plays a significant role in the chemotherapeutic drug resistance of tumors. In our study, the downregulation of NR5A2 expression resulted in lower cell viability among the cSCC cells treated with cisplatin; as well as an increase in the proportion of apoptotic cells and a decrease in the clone formation ability. These results indicate that the inhibition of NR5A2 expression leads to an enhanced inhibition of cisplatin to cSCC cells. Both CCK‐8 experiments and clone formation experiments confirmed that the knockdown of NR5A2 had a significant inhibitory effect on the proliferative ability of cells, and in clone formation experiments, cisplatin had a stronger clone‐forming ability on cells than the sh‐NR5A2 group, which suggests that the effect of NR5A2 may be more effective than that of cisplatin in inhibiting the proliferation of cancer cells, and of course, this may or may not have been due to a single setting of cisplatin's concentration results. Although both the CCK‐8 and clone formation assays confirmed that the knockdown of NR5A2 had a significant inhibitory effect on the proliferative ability of the cSCC cells, the clone formation assay revealed that the cisplatin‐treated cSCC cells showed better clone formation abilities compared to the NR5A2 silencing group, suggesting that NR5A2 silencing may be a more effective strategy than cisplatin treatment in inhibiting the proliferation of cancer cells. Certainly, it may be due to a single setting of concentration of cisplatin results, further studies are also very meaningful. In our study, the additive effect of sh‐NR5A2 combined with cisplatin on the apoptosis rate was lesser than the individual therapeutic effects of the two agents acting separately. This is probably because a slight change in the expression level of NR5A2, which is a transcription factor, may lead to a cascade effect activating its downstream target genes, while cisplatin, on the other hand, prevents DNA replication. Although the regulatory relationship between NR5A2 and the targets of cisplatin is unknown, we speculate that it may contribute to the above result.

Although we used clinical samples for the analysis of NR5A2 expression levels in cSCC cells, the small sample size of the study served as its major limitation. However, since we have examined NR5A2 expression in cSCC cells at the cellular level, we believe that our findings are valuable in understanding the functions of NR5A2 in cSCC. In the future, we aim to collect more clinical samples and refine our study further to facilitate additional analysis.

## 5 CONCLUSION

This study demonstrated that NR5A2 knockdown in cSCC cells inhibits the malignant behavior of the cells. Additionally, NR5A2 knockdown also disrupts EMT, blocks the cell cycle at the G0/G1 phase, and enhanced inhibition of proliferation and promotion of apoptosis by cisplatin treatment. These experiments provide evidence that NR5A2 plays an integral role in the development and progression of cSCC.

## AUTHOR CONTRIBUTIONS

**Wang Ye**: Data curation; formal analysis; funding acquisition; methodology; writing—original draft. **Cao Ya‐xuan**: Data curation; formal analysis; methodology; validation. **Tang Shan‐shan**: Data curation; formal analysis; methodology. **Long Qiu**: Data curation; formal analysis; methodology. **Ma Ting**: Data curation; validation. **Chen Shao‐jie**: Data curation; resources; supervision; writing—original draft. **Cao Yu**: Conceptualization; writing—review and editing.

## CONFLICT OF INTEREST STATEMENT

The authors declare no conflict of interest.

## Data Availability

The data that support the findings of this study are reported in the manuscript and Supporting Information.

## References

[1] Chang MS , Azin M , Demehri S . Cutaneous squamous cell carcinoma: the frontier of cancer immunoprevention. Annu Rev Pathol Mech Dis. 2022;17:101‐119. 10.1146/annurev-pathol-042320-120056 35073167

[2] Gross ND , Miller DM , Khushalani NI , et al. Neoadjuvant cemiplimab for stage II to IV cutaneous squamous‐cell carcinoma. N Engl J Med. 2022;387(17):1557‐1568. 36094839 10.1056/NEJMoa2209813 PMC9844515

[3] Stratigos AJ , Garbe C , Dessinioti C , et al. European interdisciplinary guideline on invasive squamous cell carcinoma of the skin: part 2. treatment. Eur J Cancer. 2020;128:83‐102. 32113942 10.1016/j.ejca.2020.01.008

[4] Fletterick R . NR5A2 discovering compounds that block tumor growth in PDAC. J Surg Oncol. 2017;116(1):89‐93. 28445593 10.1002/jso.24639 PMC5519325

[5] Xiao L , Wang Y , Xu K , et al. Nuclear receptor LRH‐1 functions to promote castration‐resistant growth of prostate cancer via its promotion of intratumoral androgen biosynthesis. Cancer Res. 2018;78(9):2205‐2218. 29438990 10.1158/0008-5472.CAN-17-2341

[6] Sandhu N , Rana S , Meena K . Nuclear receptor subfamily 5 group A member 2 (NR5A2): role in health and diseases. Mol Biol Rep. 2021;48(12):8155‐8170. 34643922 10.1007/s11033-021-06784-1

[7] He T , Shen H , Wang S , et al. MicroRNA‐3613‐5p promotes lung adenocarcinoma cell proliferation through a RELA and AKT/MAPK positive feedback loop. Mol Ther Nucl Acids. 2020;22:572‐583. 10.1016/j.omtn.2020.09.024 PMC7562961 33230458

[8] Ye T , Li J , Sun Z , et al. Nr5a2 promotes cancer stem cell properties and tumorigenesis in nonsmall cell lung cancer by regulating Nanog. Cancer Med. 2019;8(3):1232‐1245. 30740909 10.1002/cam4.1992 PMC6434341

[9] Qian L , Liang Z , Wang Z , et al. Cellular gp96 upregulates AFP expression by blockade of NR5A2 SUMOylation and ubiquitination in HCC. J Mol Cell Biol. 2023;15(5):027‐041. 10.1093/jmcb/mjad027 PMC10748477 37204028

[10] Chand AL , Wijayakumara DD , Knower KC , et al. The orphan nuclear receptor LRH‐1 and ERalpha activate GREB1 expression to induce breast cancer cell proliferation. PLoS One. 2012;7(2):e31593. 22359603 10.1371/journal.pone.0031593 PMC3281101

[11] Pang JMB , Molania R , Chand A , et al. LRH‐1 expression patterns in breast cancer tissues are associated with tumour aggressiveness. Oncotarget. 2017;8(48):83626‐83636. 29137369 10.18632/oncotarget.18886 PMC5663541

[12] Seacrist CD , Kuenze G , Hoffmann RM , et al. Integrated structural modeling of full‐length LRH‐1 reveals inter‐domain interactions contribute to receptor structure and function. Structure. 2020;28(7):830‐846.e9. 32433991 10.1016/j.str.2020.04.020 PMC7347456

[13] Meinsohn MC , Smith OE , Bertolin K , Murphy BD . The orphan nuclear receptors steroidogenic factor‐1 and liver receptor homolog‐1: structure, regulation, and essential roles in mammalian reproduction. Physiol Rev. 2019;99(2):1249‐1279. 30810078 10.1152/physrev.00019.2018

[14] Yang Q , Deng L , Li J , Miao P , Liu W , Huang Q . NR5A2 promotes cell growth and resistance to temozolomide through regulating notch signal pathway in glioma. Onco Targets Ther. 2020;13:10231‐10244. 33116604 10.2147/OTT.S243833 PMC7567570

[15] Qiao J , Chen Y , Mi Y , et al. NR5A2 synergizes with NCOA3 to induce breast cancer resistance to BET inhibitor by upregulating NRF2 to attenuate ferroptosis. Biochem Biophys Res Commun. 2020;530(2):402‐409. 32536370 10.1016/j.bbrc.2020.05.069

[16] Cobo I , Martinelli P , Flández M , et al. Transcriptional regulation by NR5A2 links differentiation and inflammation in the pancreas. Nature. 2018;7693:533‐537. 10.1038/nature25751 PMC6121728 29443959

[17] von Figura G , Morris JP , Wright CVE , et al. Nr5a2 maintains acinar cell differentiation and constrains oncogenic Kras‐mediated pancreatic neoplastic initiation. Gut. 2013;63(4):656‐664. 23645620 10.1136/gutjnl-2012-304287 PMC3883808

[18] Gkikas D , Stellas D , Polissidis A , et al. Nuclear receptor NR5A2 negatively regulates cell proliferation and tumor growth in nervous system malignancies. Proc Natl Acad Sci USA. 2021;118(39):e243118. 10.1073/pnas.2015243118 PMC8488649 34561301

[19] Flandez M , Cendrowski J , Cañamero M , et al. Nr5a2 heterozygosity sensitises to, and cooperates with, inflammation in KRas(G12V)‐driven pancreatic tumourigenesis. Gut. 2014;63(4):647‐655. 23598351 10.1136/gutjnl-2012-304381

[20] Nadolny C , Dong X . Liver receptor homolog‐1 (LRH‐1): a potential therapeutic target for cancer. Cancer Biol Ther. 2015;16(7):997‐1004. 25951367 10.1080/15384047.2015.1045693 PMC4622691

[21] Farooqi AA , Mukhanbetzhanovna AA , Yilmaz S , Karasholakova L , Yulaevna IM . Mechanistic role of DANCR in the choreography of signaling pathways in different cancers: spotlight on regulation of Wnt/β‐catenin and JAK/STAT pathways by oncogenic long non‐coding RNA. Non‐coding RNA Res. 2021;6(1):29‐34. 10.1016/j.ncrna.2021.01.001 PMC7851422 33553855

[22] Liu L , Li Y , Pan B , et al. Nr5a2 promotes tumor growth and metastasis of gastric cancer AGS cells by Wnt/beta‐catenin signaling. Onco Targets Ther. 2019;12:2891‐2902. 31114234 10.2147/OTT.S201228 PMC6489909

[23] Bayrer JR , Mukkamala S , Sablin EP , Webb P , Fletterick RJ . Silencing LRH‐1 in colon cancer cell lines impairs proliferation and alters gene expression programs. Proc Natl Acad Sci USA. 2015;112(8):2467‐2472. 25675535 10.1073/pnas.1500978112 PMC4345603

[24] Benod C , Carlsson J , Uthayaruban R , et al. Structure‐based discovery of antagonists of nuclear receptor LRH‐1. J Biol Chem. 2013;288(27):19830‐19844. 23667258 10.1074/jbc.M112.411686 PMC3707686

[25] Guo F , Zhou Y , Guo H , Ren D , Jin X , Wu H . NR5A2 transcriptional activation by BRD4 promotes pancreatic cancer progression by upregulating GDF15. Cell Death Discov. 2021;7(1):78‐93. 33850096 10.1038/s41420-021-00462-8 PMC8044179

[26] Pulford CS , Uppalapati CK , Montgomery MR , Averitte RL , Hull EE , Leyva KJ . A hybrid epithelial to mesenchymal transition in ex vivo cutaneous squamous cell carcinoma tissues. Int J Mol Sci. 2022;23(16):9183‐9199. 36012449 10.3390/ijms23169183 PMC9408944

[27] Li X , Zhao S , Bian X , et al. Signatures of EMT, immunosuppression, and inflammation in primary and recurrent human cutaneous squamous cell carcinoma at single‐cell resolution. Theranostics. 2022;12(17):7532‐7549. 36438481 10.7150/thno.77528 PMC9691356

[28] Luo Z , Li Y , Zuo M , et al. Effect of NR5A2 inhibition on pancreatic cancer stem cell (CSC) properties and epithelial‐mesenchymal transition (EMT) markers. Mol Carcinog. 2017;56(5):1438‐1448. 27996162 10.1002/mc.22604 PMC5392129

[29] Wang S , Zou Z , Luo X , Mi Y , Chang H , Xing D . LRH1 enhances cell resistance to chemotherapy by transcriptionally activating MDC1 expression and attenuating DNA damage in human breast cancer. Oncogene. 2018;37(24):3243‐3259. 29545602 10.1038/s41388-018-0193-4

[30] Jin J , Jin J , Woodfield SE , et al. Targeting LRH‑1 in hepatoblastoma cell lines causes decreased proliferation. Oncol Rep. 2019;41(1):143‐153. 30320362 10.3892/or.2018.6793 PMC6278492

