# Correction to “NR5A2 Promotes Malignancy Progression and Mediates the Effect of Cisplatin in Cutaneous Squamous Cell Carcinoma”

**DOI:** 10.1002/iid3.70125

**Published:** 2025-01-14

W. Ye, C. Ya‐Xuan, T. Shan‐Shan, L. Qiu, M. Ting, C. Shao‐Jie, and C. Yu, “NR5A2 Promotes Malignancy Progression and Mediates the Effect of Cisplatin in Cutaneous Squamous Cell Carcinoma,” *Immunity, Inflammation and Disease* 12, no. 2 (2024): e1172, https://doi.org/10.1002/iid3.1172.

In the original version of this paper, the images in the wound healing experiment for two cell lines were inadvertently duplicated in Figure 4D. The authors have since reviewed the original scans and repeated the experiment. The figures have been corrected accordingly, and this revision does not impact the conclusions of the article in any way. The corrected Figure 4 is shown below. We apologize for this error.

**Figure 4:**
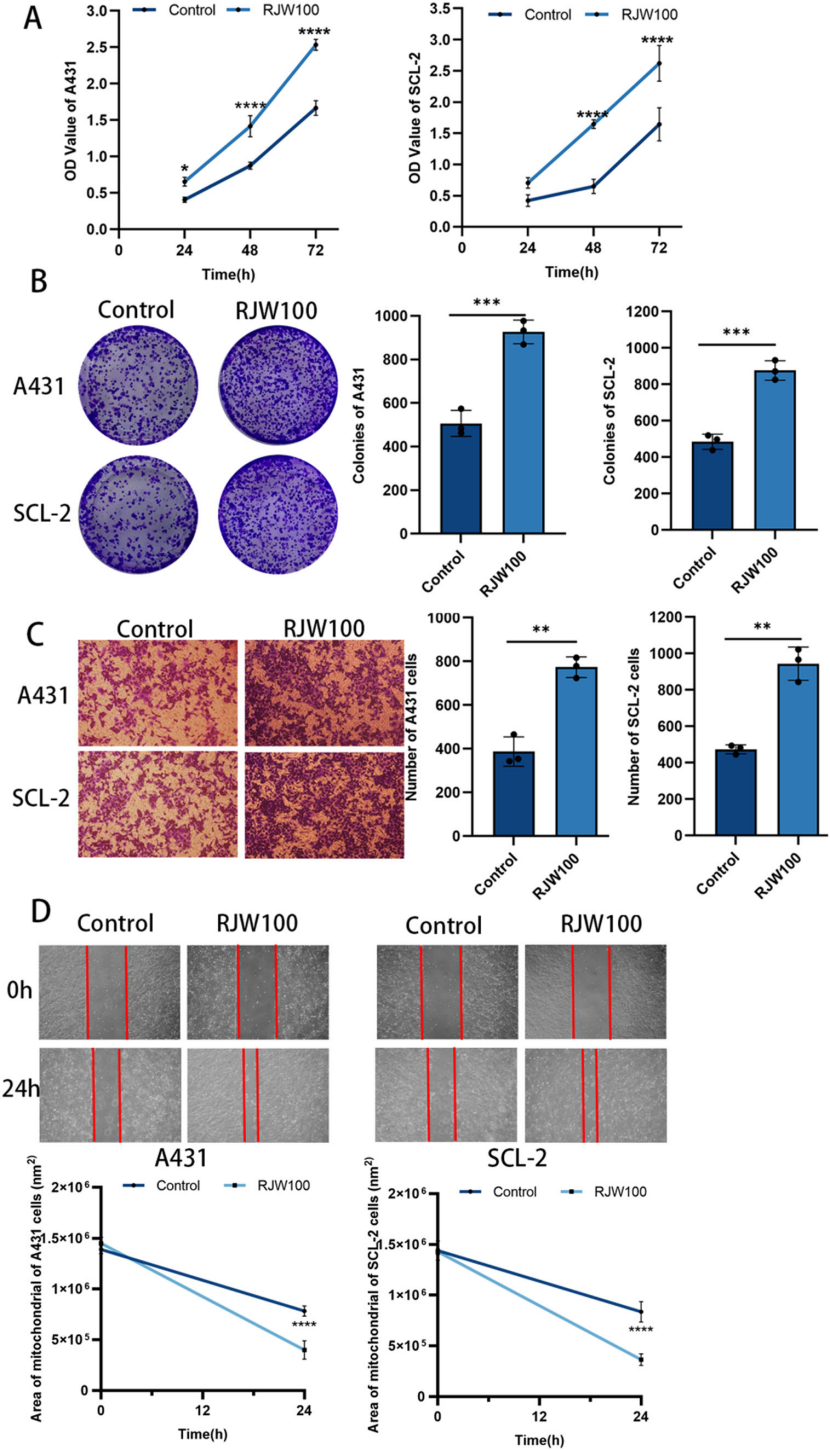
.

